# Do Fitter Children Better Assess Their Physical Activity with Questionnaire Than Less Fit Children?

**DOI:** 10.3390/ijerph19031304

**Published:** 2022-01-24

**Authors:** Jerneja Premelč, Kaja Meh, Henri Vähä-Ypyä, Vedrana Sember, Gregor Jurak

**Affiliations:** 1 Faculty of Sport, University of Ljubljana, 1000 Ljubljana, Slovenia; kaja.meh@fsp.uni-lj.si (K.M.); vedrana.sember@fsp.uni-lj.si (V.S.); gregor.jurak@fsp.uni-lj.si (G.J.); 2 UKK-Institute, 33500 Tampere, Finland; Henri.Vaha-Ypya@ukkinstituutti.fi

**Keywords:** assessment, physical fitness, accelerometer, validity, reliability, youth

## Abstract

Most physical activity (PA) questionnaires assess moderate to vigorous PA (MVPA) describing the physical exertion of individuals that might be influenced by their physical fitness. Therefore, the aim of this study was to determine whether fitter children could better assess their PA with the questionnaire than less fit children. The cross-sectional validation study was conducted with 108 children (60 girls) aged 11 to 14 years, who were divided into three fitness groups based on the results of the 600 m running test. To answer the research question, the agreement between their assessment of PA using the SHAPES questionnaire and the UKK RM42 accelerometer data was analysed. One quarter of the participants achieved at least 60 min of MVPA each day, measured by accelerometer. The average MVPA obtained was 97.8 ± 35.6 min per day, with the high fitness group having a significantly higher value compared with the other groups. Moderate to high validity coefficients were found in the high fitness group (Spearman’s ρ range 0.34–0.70). In contrast, the lower fitness groups had poor to moderate validity for all variables (Spearman’s ρ range 0.03–0.42). These results suggest that the fittest children self-assess their PA with the questionnaire better than less fit children, which may advance new directions for the development and evaluation of PA questionnaires and their usability.

## 1. Introduction

Sufficient levels of physical activity (PA) are associated with various health benefits for children and adolescents in terms of physical, psychological, social, and cognitive health [1]. Although recent research shows that even low intensity PA (LPA) is associated with health benefits in adults and adolescents [2], higher PA intensities are associated with more health benefits [3,4]. Consistent with this evidence, the World Health Organization (WHO) recommends at least 60 min of moderate to vigorous PA (MVPA) daily for children and adolescents (5–17 years) to achieve adequate health benefits [5]. Further, movement behaviour in combination with nutrition habits determine physical fitness, which is the best indicator of health in childhood and adolescence [6].

Physical fitness is defined as the ability to perform daily tasks with vigour and alertness without excessive fatigue and to summon sufficient energy for leisure activities and unforeseen emergencies. It encompasses a wide range of physical abilities, such as cardiorespiratory fitness, strength, coordination, and flexibility. Various fitness test batteries are used for epidemiological assessment of physical fitness in children and adolescents, e.g., Eurofit [7], AAHPER Youth Fitness Project [8], The President’s Challenge [9], Fitnessgram [8,10], Japanese MEXT Fitness Test [11], The International Physical Fitness Test [12], ALPHA Fitness Test Battery [13], SLOfit Test Battery [14].

Further, several methods can be used to determine the amount and intensity of children’s PA. Accelerometers are quite valid assessment tools, but they are less suitable for large epidemiological studies. Therefore, PA questionnaires are a good choice, but they should be used with caution with children, as their understanding of the PA concept is still developing. [15]. The use of self-report PA questionnaires is not appropriate for children under 10 years of age [16]; therefore, parents are asked to report the child’s data on PA. However, there are several different PA questionnaires for children and adolescents whose measurement characteristics vary slightly. Most of them rely on recollection of a past or a usual week’s movement behaviour, and descriptions of physical responses to PA are used to distinguish PA intensities. The descriptions are based on reactions to physical exertion, such as heavy breathing, increased heartbeat, and sweating, and descriptions are highly subjective. Such descriptions are supported by some possible examples of PA. A recent meta-analysis analysing the results of 20 different PA questionnaires for children and adolescents found low average validity results (moderate to vigorous PA (MVPA) = 0.27, moderate PA (MPA) = 0.24, vigorous PA (VPA) = 0.33) and overall moderate to high reliability coefficients (MVPA = 0.75, MPA = 0.56, VPA = 0.68) [17]. The School Health Action, Planning, and Evaluation System (SHAPES) questionnaire [18] is one of the available PA questionnaires, designed for large-scale data collection efforts in schools, which was rated as one of the top five questionnaires measuring physical activity in youth (Biddle et al., 2011). Its reliability and validity results are comparable with other questionnaires; moreover, it is quite interesting for children to complete it since it provides a daily hours and minutes scale to report their daily PA (see Supplementary File S1 (Q3, Q4)). This enables children to report their PA for each day of the past week separately, which makes recall easier and quicker. MPA and VPA are assessed separately, and screen time questions are also included. 

Previous studies have shown differences in the validity and reliability of PA questionnaires between genders in favour of boys [19,20,21], as well as according to body mass index (BMI) [20], where higher validity was reported in under and normal weight groups of children [22]. These differences may not occur merely as a result of gender and BMI as such but could be affected by differences in physical fitness in genders and different BMI groups. Namely, PA questionnaires assess MPA and VPA by describing individuals’ physical exertion. Thus, respondents’ physical fitness level could affect the way they perceive and evaluate their PA. However, based on our knowledge, no study has been conducted that has analysed whether fitter individuals better assess their PA with a questionnaire than less fit individuals. Therefore, we prepared a study to test the reliability and validity of the SHAPES questionnaire with a hip-worn accelerometer between three differently fit groups of children to analyse this hypothesis.

## 2. Materials and Methods

### 2.1. Participants

**Figure 1:**
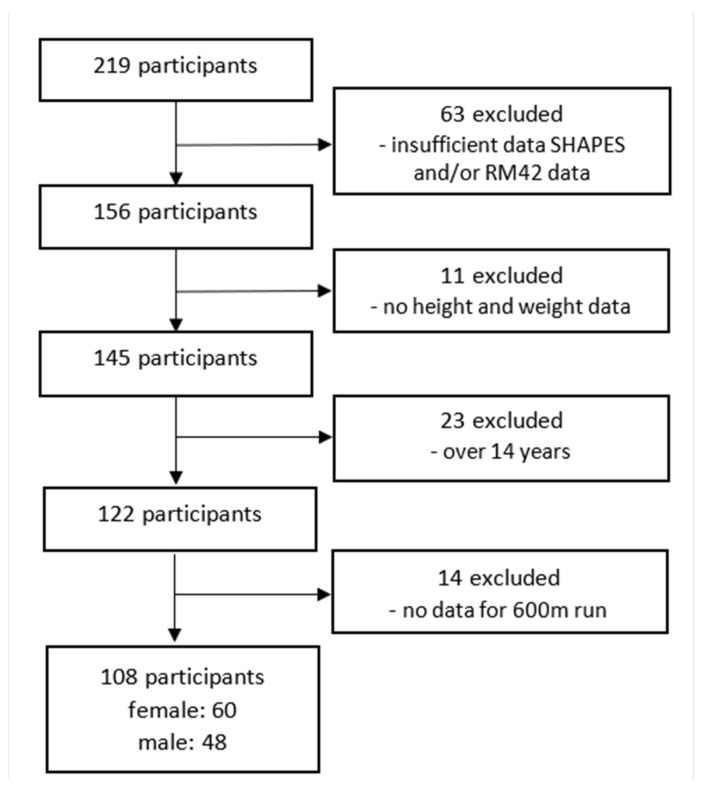
Participant flow diagram.

We recruited our initial sample with the help of school coordinators who were physical education teachers at 9 Slovenian elementary schools in semi-rural and urban settings. The school coordinators prepared a meeting for the parents of the 11- to 14-year-old children and, together with the researchers, introduced them to the study design and purpose and invited them to participate in the study. Parents or guardians provided written informed consent for all participating children. Only healthy participants were included in the study. The initial sample consisted of 219 children (119 girls) who participated in the EUPASMOS project (described in detail in [23]) that took place from October till November 2018. Ethical permission was obtained from the Faculty of Sport in Ljubljana in accordance with the Declaration of Helsinki (No: 2020-274). Due to invalid or missing data, we excluded 111 participants from the sample and included 108 children (48 boys and 60 girls) in the analysis (Figure 1). We found no differences in BMI between the 108 children included in the analysis and children who were excluded (*t(197)* = 2.02, *p* = 0.45) due to different reasons. 

### 2.2. Measurement

Children participated in the study at two time points 1 week apart (test–retest reliability study). At the first measurement time point, children completed an online version of the PA questionnaire and began wearing the accelerometer. They were instructed to behave as they would in a normal week and to wear the accelerometers all the time for the next seven consecutive days, except during water activities (e.g., showering, swimming). A researcher showed them how to wear and apply the accelerometer during daily activities (on the hip) and at night (on the wrist), and also gave them written instructions on how to wear it properly (see Supplementary File S2) so that they would be prepared to care for the device themselves. After one week, the participants returned for the second visit. They returned the accelerometers and took anthropometric measurements. Finally, they completed the PA questionnaire (for the test–retest study). In addition, cardiorespiratory fitness data were collected as part of the annual SLOfit testing, the Slovenian system for monitoring children’s physical fitness [14]. 

There were school holidays during the data collection, but the children did not participate in the study during that week and the week after the holidays, as their physical activity changes during holidays [24]. 

#### 2.2.1. Self-Reported PA

Self-reported PA was measured using a Slovene version of the 3 items from the SHAPES questionnaire [25], which was back-translated to Slovene language, following WHO translation protocol [26]. The SHAPES questionnaire includes a last 7-days recall tool in which children rate the amount of time they spent on MPA, VPA, and screen activities using a special scale. For each day of the week, they report the time spent being physically active or spending time before the screen in hours and minutes (with 15 min intervals) (see Supplement File S1 for the SHAPES questionnaire). Daily time of PA can be used later to check whether children are sufficiently active (at least 60 min of MVPA per day), according to guidelines from WHO [5]. To better understand MPA and VPA, the questions include a description of physiological responses common for the selected PA intensity and examples of typical activities for each intensity of PA. MPA was described as “lower-intensity physical activities such as brisk walking, bicycling, in-line skating, and other activities that increase your breathing,” while VPA was described as “team sports, fast dancing, jumping rope, and any other physical activity that significantly increases your heart rate and makes you breathe hard and sweat.” Original version of SHAPES has moderate test–retest reliability (mean kappa coefficient = 0.57) and moderate criterion validity for MVPA (Spearman’s ρ = 0.44) and low validity results for MPA (Spearman’s ρ = 0.31) and VPA (Spearman’s ρ = 0.25) [25]. 

#### 2.2.2. Accelerometer Measured PA

A tri-axial accelerometer (RM42, UKK Terveyspalvelut Oy, Tampere, Finland) was worn on the right hip during waking hours and on the non-dominant wrist during time in bed. The acceleration data were collected within a range of ± 16 G at a sampling rate of 100 Hz and stored on a hard disk for further analysis. The analysis of PA was based on the mean amplitude deviation (MAD) in six-second epochs [27]. For each epoch, the MAD values were converted to METs (3.5 mL/kg/min of oxygen consumption). The epoch-wise MET values were further smoothed by calculating exponential moving average for each epoch time point [28]. MAD has been validated against ActiGraph; validity for sedentary behaviour was strong (ICC = 0.992) and moderate for MVPA (ICC = 0.366) [29].

The smoothed data were analysed in 6 s epochs, and PA cut points were set as follows: 3.0 METs ≤ MPA < 6.0 METs and VPA ≥ 6.0 METs. A valid day was defined as having at least 600 min of monitor wear and at least 4 valid wear days, one of which had to be weekend day. Only participants with sufficient accelerometer data were included in the study.

#### 2.2.3. Anthropometry 

Height (to the nearest 0.1 cm) and weight (to the nearest 0.1 kg) were measured using Seca 799 electronic scales (Seca Germany, Hamburg, Germany) while participants were without shoes and in light clothing. We calculated BMI from height and weight and divided participants into three categories based on WOF criteria: underweight, normal weight, and overweight [30]. 

#### 2.2.4. Cardiorespiratory Fitness

The 600 m run was measured to the second. This test has been used for more than 30 years in the SLOfit test battery to assess the cardiorespiratory fitness of children in all elementary and secondary schools as part of the Slovenian national physical fitness monitoring system, called SLOfit [31]. The fitness test was developed back in the 1980s based on a comprehensive study of various motor tests [14].

### 2.3. Statistical Analysis

Statistical analyses were undertaken using SPSS V.21.0 software (SPSS Inc., Chicago, IL, USA). The participants were stratified by fitness level into three groups using terciles of standardized results of 600 m run test: high fit (HF), intermediate fit (IF), and low fit (LF) group. Descriptive analyses were carried out for all variables. Two-way ANOVAs with post hoc Tukey’s tests were used to determine differences between fit groups for age, BMI, MPA, VPA, and MVPA. Time spent performing MVPA was calculated by summing the weekly time spent performing MPA and VPA. Mean difference score was calculated for MPA, VPA, and MVPA as data from the questionnaire minus the accelerometer value. Statistical significance was accepted at *p* < 0.05.

The PA variables used to assess the reliability and validity of the SHAPES were total time spent on MPA, VPA, and MVPA intensity level. Test–retest reliability was evaluated by the parametric interclass correlation coefficient (ICC) with 95% confidence interval and Cronbach’s alpha. ICCs higher than 0.7 were considered as acceptable reliability [32]. The validity assessed by non-parametric Spearman’s rank correlation coefficient was used to evaluate the correlation between PA (minutes per day) gathered by the SHAPES questionnaire and the RM42 accelerometer data. Spearman’s ρ estimates, values lower than 0.29, between 0.3 and 0.49, between 0.5 and 0.69, between 0.7 and 0.89, and greater than 0.9 are indicative of very low, low, moderate, high and very high validity, respectively [33].

## 3. Results

Characteristics of 108 participants (55.6% girls) in this study are presented in Table 1. No statistically significant differences between boys and girls within groups were noted in fitness results (LF *p* = 0.937, IF *p* = 0.722, HF *p* = 0.064). Therefore, further analysis was carried out for both sexes together. The fitness groups did not differ based on age (*p*p = 0.32) and BMI (*p* = 0.07). The average age was 12 ± 1.1 years, and the majority of participants (60.2%) were classified in the normal body weight category. The HF group had the most underweight children (46.3%) compared with the other two fit groups. More detailed characteristics are described in Table 1. 

On average, it took participants 123 s to complete selected three items of the SHAPES questionnaire. They wore the accelerometer for at least 600 min to participate in the study; no differences in wearing time were found between fitness groups. PA intensity levels in Table 2 are presented based on the SHAPES (e.g., Q_MVPA) and accelerometer data (e.g., AC_MVPA). 

Only 27 children (25%) had at least 60 min of MVPA each day measured by accelerometers. A higher percentage of MVPA of at least 60 min each day was in the HF group (31.7%) compared with LF (22.2%) and IF group (20%). On the other hand, the average MVPA was 97.8 ± 35.6 min per day, with significant differences between all fitness groups.

Based on the questionnaire, the HF group reported the highest amount of time being physically active (130 ± 50 min per day). However, there were no significant differences in self-reported physical activity between fitness groups. The total number of minutes per day spent on MVPA estimated with the SHAPES questionnaire was 20.3% higher than that obtained with the RM42 accelerometer. The mean difference value for MVPA was 19.6 ± 54.3 min per day. The largest differences between the SHAPES questionnaire and RM42 accelerometer data were for VPA (298%). The HF group reported significantly more time spent on VPA than the LF (*p* = 0.001) and IF groups (*p* = 0.005). Overall, MPA was reported 38.8% lower than the RM42 accelerometers. All groups reported higher numbers of MVPA and VPA with the SHAPES questionnaire than measured with the RM42 accelerometers. In contrast, the SHAPES questionnaire showed lower values for MPA in all groups compared with the RM42 accelerometers.

The test–retest reliability of the SHAPES questionnaire is presented in Table 3. Interclass correlation coefficients (ICCs) and Cronbach’s alpha were calculated for different PA levels for each fitness group. The ICC ranged from 0.41 to 0.86 with wide 95% confidence intervals. The highest reliability was found for MVPA (ICC = 0.75; α = 0.75). The LF group had higher ICC in MVPA and VPA compared with the other groups, while the IF group had weak reliability in all variables, especially in VPA (ICC = 0.41; α = 0.41, *p* = 0.053).

Spearman’s ρ generally showed low validity for most variables. Only in the HF group was moderate validity found for MVPA (Spearman’s ρ = 0.50) and high validity for VPA (Spearman’s ρ = 0.70). Very low and low validity were found in LF (Spearman’s ρ = 0.03–0.14) and IF group (Spearman’s ρ = 0.12–0.42), respectively (Table 4).

**Figure 2:**
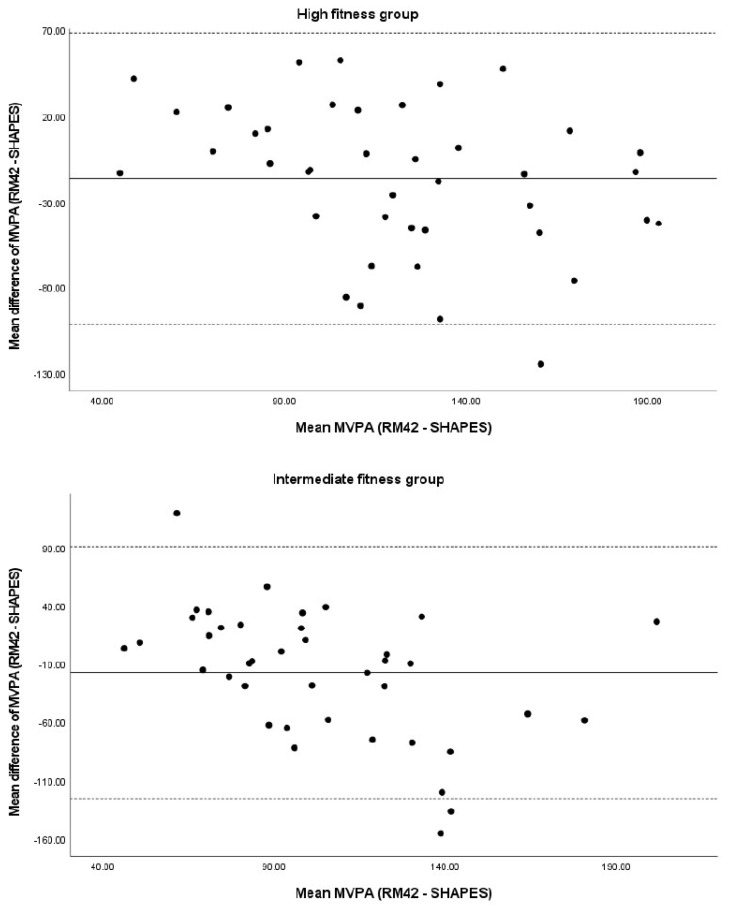

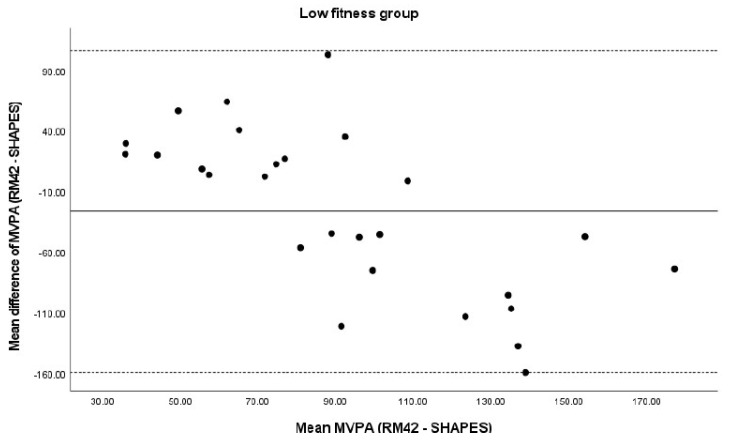
Bland–Altman plots of the difference in MVPA between UKK RM42 accelerometer and SHAPES questionnaire (min/day) with 95% limit of agreement.

To compare the pattern of reporting PA between fitness groups, we constructed a Bland–Altman diagram (Figure 2). Overreporting of MVPA with SHAPES was lowest for HF (−16.6 min ± 43.3), whereas participants from IF (−17.9 min ± 55.3) and LF (−26.6 min ± 67.8) showed greater differences between the two methods. In all groups, participants both under- and over-reported MVPA, but in the IF and LF groups, the more physically active participants overestimated MVPA more often, whereas the less active participants underestimated their PA more often. 

The visually noted trend of PA self-reporting by the fitness groups showed differences between them. The self-reporting of PA was more consistent with accelerometers measurements among participants in the HF group than in the other two groups. This was reflected in the higher validity of the SHAPES questionnaire in the HF group.

## 4. Discussion

The present study is the first to investigate whether children’s level of fitness affects self-reported PA with a questionnaire, since PA questionnaires are based on descriptions of physical responses to PA, which are highly subjective and could depend on the individual’s fitness. The reliability and validity of the SHAPES questionnaire between three differently fit groups of 108 children classified on the basis of a 600 m running test were used to analyse the problem. The main finding is that the SHAPES questionnaire appears to be more valid for assessing MVPA in the fittest children, with moderate criterion validity (Spearman’s ρ = 0.50). The second important finding is that all fit groups of children underestimated MPA and overestimated VPA when using the SHAPES questionnaire compared with the RM42 accelerometer data. Overall, our results suggest that the Slovenian version of the SHAPES questionnaire has acceptable test–retest reliability in children aged 11–14 years, while its validity is low.

MVPA is the most popular PA category in science and policy making since it encompasses a wide range of health-promoting movements. Since there are some peculiarities associated with assessing VPA in selected algorithms (described below), we focused our discussion of the validity of the SHAPES questionnaire on the MVPA construct. The lowest mean difference score between the questionnaire and accelerometer data on MVPA, expressed as a percentage, was found in the HF group. The criterion validity of the MVPA data from the SHAPES questionnaire in the HF group, which was based on the UKK RM42 accelerometer data, showed moderate validity (Spearman’s ρ = 0.50), while the validity in the other two groups was very low (Spearman’s ρ = 0.20 for the IF group and 0.14 for the LF group). The validity results in our study were much lower than in the original validation study of this questionnaire using the ActiGraph AM7164 as a reference criterion [25]. Namely, the Spearman’s ρ in the original study was 0.44, whereas in our study it was 0.32 for the whole sample. 

Since this is the first investigation of how individual fitness level can alter self-reported PA, the results can only be compared with studies that have included variables that might affect the variability of fitness. For example, boys tend to have higher fitness compared with girls, and previous research has shown that criterion validity in a group of boys (r = 0.38, *p* < 0.01) is higher than in girls (r = 0.26, *p* < 0.05) [19]. Similarly, BMI is associated with fitness level—namely, overweight and obese children have lower level of physical fitness [34,35,36]. In accordance with such findings, higher validity was reported in underweight and normal weight groups of children (0.36, *p* < 0.01; obese children = 0.17, *p* > 0.05) [22], where correlations between a PA questionnaire and accelerometer were higher in the non-obese group (0.47, *p* = 0.01) compared with the obese group (0.14, *p* = 0.24) [37]. 

Considering the described findings from our and other related studies as well as the limitations of our study, described further, our results suggest that fitter children are better at self-assessing their PA with the questionnaire than less fit children. Indeed, the descriptions of MVPA used in most PA questionnaires are based on individual perceptions of physiological responses to PA, such as heavy breathing, faster heartbeat, and sweating. Therefore, less fit children perceive such responses at lower METs than fitter children because their cardiorespiratory fitness is lower compared with fitter children. Such findings advocate that: (a) when checking the validity of the PA questionnaires, the structure of the sample should be controlled according to the fitness of the participants—as an alternative, self-reported BMI could be collected as a less reliable indicator of fitness; (b) the results of the PA questionnaire on individual level should be interpreted according to the fitness of the individual—in our study, HF children over-reported MVPA by 15%, while LF children for over-reported by 34%.

According to the recommendations of WHO [5], only 25% of children were physically active for at least 60 min each day, noted by the UKK RM42 accelerometer, while 45% were assessed by the SHAPES questionnaire. Therefore, the misclassification of children who reached the recommendations of WHO was higher in our study than in a recent study conducted in Croatia using the same questionnaire but a different device—the SenseWear wristband [38]. There, the difference between SHAPES and the accelerometer was 1.8% for boys and 14.2% for girls. This large discrepancy could be due to the different devices and location of wearing the devices (upper arm vs. hip) in the two studies.

Moreover, the average MVPA obtained with the UKK RM42 accelerometer in our study was 97.8 min per day, which is high compared with other studies that obtained average MVPA values ranging from 37.3 to 57.6 min per day [39,40]. This could be due to the fact that in our study the cut-off value for MPA was set at 3 MET, which could lead to an overestimation of MVPA in adolescents [41]. Hence, many of accelerometers’ studies on children use 4 MET as cut point for MPA [42]. Nevertheless, the expected trend of the difference in assessing MPA and VPA with the questionnaire was found. Namely, we found that all fit groups of children underestimated MPA and overestimated VPA with the SHAPES questionnaire compared with the UKK RM42 accelerometer data. This is a systematic misclassification of moderate activity as vigorous, which has also been found in other studies [25,43,44]. While the MPA differences were in the expected range, the VPA differences were really large, e.g., 11 min measured with UKK RM42 and 64 min reported with SHAPES questionnaire overall.

Considering that the VPA threshold was set at 6 MET and above, these discrepancies again point to some peculiarities of assessing PA with the MAD algorithm. In particular, two recent validation studies in the adult population using the UKK RM42 and the MAD algorithm also reported low VPA values. One of the studies reported an average weekly time of 5 min [45] and the other 22 min per week spent in VPA [46]. However, the MAD algorithm has shown a very strong association with VO_2_ during ambulatory activities [47], so further investigation of the MAD algorithm in practise is needed. In addition, other accelerometer algorithms have also been found to have limitations in assessing VPA [48,49,50]. Nonetheless, accelerometers still have several advantages over PA questionnaires [51,52]. However, when validating PA questionnaires with accelerometers, the measurement characteristics of their algorithms should be considered in comparison with the gold standard.

Test–retest reliability of the Slovenian version of the SHAPES questionnaire assessed moderate results, with the highest ICC values for MVPA (0.75). These results are similar to other studies, which reported ICC values ranging from 0.64 to 0.92 [39,53]. However, it is interesting that the IF group had a really lower reliability coefficient in VPA (ICC = 0.41) than the other two fitness groups (ICC = 0.83 for LF and 0.59 for HF). We can assume that children with intermediate fitness do not have a regular VPA pattern, so their VPA could vary from week to week. In contrast, the LF and HF groups might be more consistent in their PA behaviour: LF group with regular low PA lifestyle and HF group participating in organised sports. 

### Strengths and Limitations

This is the first study to assess the reliability and validity of the PA questionnaire in children with different fitness levels. Previous studies have evaluated PA questionnaires comparing gender, different age, and BMI groups, but none reported agreement between PA questionnaires and accelerometers in different fitness groups. Nevertheless, the results of this study should be considered in light of some limitations. First, participants were divided into three groups based on the results of the 600 m run (aerobic) test, using terciles according to our sample, not as an absolute criterion. Therefore, the classification of participants into fitness categories depended on the characteristics of the sample. Previous findings suggest that Slovenian children have better cardiorespiratory fitness compared with their international peers [54]. Therefore, higher variability in fitness results, which is more common in other countries, might better indicate differences in validity of the PA questionnaires according to different fitness levels [55]. Second, due to the specifics of data collection with the UKK RM42 accelerometers, thresholds of 3.0 METs ≤ MPA < 6.0 METs and VPA ≥ 6.0 METs were used in the EUPASMOS study. This could lead to overestimation of MPA in our study, but not VPA. The third limitation is associated with the MAD algorithm for the analysis of accelerometer data, which is validated for bipedal activities. Therefore, the intensity of activities of other types, such as cycling, is likely to be underestimated. As a result, the volume of VPA might also be underestimated. However, similar problems with the measurement of VPA have been highlighted in other studies that used other algorithms for accelerometer data [48,49,50]. 

## 5. Conclusions

Our study showed that the fittest children assessed their PA more validly and differentiated better between different PA intensities according to the SHAPES questionnaire constructs. Therefore, the measurement characteristics of PA questionnaires for children and adolescents might be influenced by the physical fitness of the participants, and this issue should be further investigated. Namely, several strategies are recommended to improve the quality, validity, and reliability of PA questionnaires, and more authors suggest improving or refining the most promising currently existing PA questionnaires than developing new instruments [56,57,58]. If future studies confirm that respondent fitness affects the validity of PA questionnaires, this could open new directions for the development and evaluation of PA questionnaires and their usability. 

## Figures and Tables

**Table 1:** Basic characteristics of different fit groups and differences among them.

Group	Low Fitness (LF) (N = 27)	Intermediate Fitness (IF) (N = 40)	High Fitness (HF) (N = 41)	Total (N = 108)
Age (years)	12.6 ± 1.1	12.2 ± 1.1	12.4 ± 1.2	12.4 ± 1.1
Girls	25 (92.6%)	23 (57.5%)	12 (29.3%)	60 (55.6%)
BMI (total)	20.7 ± 3.8	19.3 ± 2.1	19.1 ± 2.1	19.6 ± 2.9
Underweight (N, % within FG)	6 (22.2%)	12 (30%)	19 (46.3%)	37 (34.3%)
Normal weight (N, % within FG)	17 (63%)	28 (70%)	20 (48.8%)	65 (60.2%)
Overweight (N, % within FG)	4 (14.8%)	0 (0%)	2 (4.9%)	6 (5.6%)

**Table 2:** Objective and subjective PA measurement of different fit groups and differences among them.

Group	Low Fitness (LF)	Intermediate Fitness (IF)	High Fitness (HF)	Total
Running 600 m (sec)	181.3 ± 22.3 ^ac^	148.9 ± 8.4 ^ac^	122.4 ± 16 ^bc^	146.9 ± 27.8
AC_MPA (min per day)	73.9 ± 25.7 ^b^	85.5 ± 26.8	97.3 ± 28.7 ^b^	87.1 ± 28.6
Q_MPA (min/per day)	55.7 ± 43.3	55.7 ± 37.3	49.4 ± 31.6	53.3 ± 36.7
MPA_Mean Difference Score (min per day)	−18.2 ± 50 ^b^ (24.6%)	−29.8 ± 47.5 (34.9%)	−47.9 ± 35.6 ^b^ (49.2%)	−33.8 ± 45.2 (38.8%)
AC_VPA (min per day)	4.5 ± 3.9	9.4 ± 9.1	16 ± 11.7	10.7 ± 10.3
Q_VPA (min per day)	49.3 ± 37.9 ^b^	57.1 ± 32 ^c^	80.4 ± 30.1 ^bc^	64 ± 35.2
VPA_Mean Difference Score (min per day)	44.8 ± 37.8 ^bc^ (796%)	47.7 ± 28.6 ^bc^ (307%)	64.5 ± 23.8 ^bc^ (203%)	53.3 ± 30.6 (298%)
AC_MVPA (min per day)	78.4 ± 27.5 ^b^	94.9 ± 32.8 ^c^	113.3 ± 36.7 ^bc^	97.8 ± 35.6
Q_MVPA (min per day)	105 ± 65.6	112.8 ± 53.2	129.9 ± 49.6	117.3 ± 55.7
MVPA_Mean Difference Score (min per day)	26.6 ± 67.8 (33.9%)	17.9 ± 55.3 (18.9%)	16.6 ± 43.3 (14.7%)	19.6 ± 54.3 (20.3%)
AC_MVPA at least 60 min each day (N, %)	6 (22.2%)	8 (20%)	13 (31.7%)	27 (25%)
Q_MVPA at least 60 min each day (N, %)	13 (48.1%)	15 (37.5%)	21 (51.2%)	49 (45.4%)

*p* < 0.05; ^a^ = significant differences between LF and IF group; ^b^ = significant differences between LF and HF group; ^c^ = significant differences between IF and HF group. Abbreviations: PA = physical activity; AC_MPA = MPA measured with accelerometer; Q_MPA = construct in questionnaire about moderate PA; AC_VPA = VPA measured with accelerometer; Q_VPA = construct in questionnaire about vigorous PA; AC_MVPA = MVPA measured with accelerometer; Q_MVPA = constructs in questionnaire about moderate and vigorous PA: % are reported within fit group; Mean Difference Score is calculated as Q minus AC value.

**Table 3:** Test–retest reliability for each PA category of the SHAPES questionnaire.

	Group	Test 1	Test 2	ICC (95% CI)	Cronbach’s Alpha
Q_MPA	LF	48.2 ± 37	55.7 ± 43.3	0.62 (0.18–0.83)	0.62 *
	IF	54.1 ± 39.6	55.7 ± 37.3	0.69 (0.4–0.84)	0.68 *
	HF	55 ± 35	49.4 ± 31.6	0.78 (0.58–0.9)	0.78 *
	Total	52.9 ± 36.8	53.3 ± 36.7	0.69 (0.55–0.79)	0.69 *
Q_VPA	LF	52.9 ± 34.2	49.3 ± 37.9	0.83 (0.62–0.92)	0.82 *
	IF	58 ± 26.5	57.1 ± 32	0.41 (0.12–0.7)	0.41
	HF	77.4 ± 31.4	80.4 ± 30.1	0.59 (0.23–0.78)	0.59 *
	Total	64.9 ± 32	64 ± 35.2	0.7 (0.54–0.79)	0.69 *
Q_MVPA	LF	101.1 ± 56.3	105 ± 65.6	0.86 (0.69–0.94)	0.86 *
	IF	112 ± 49.8	112.8 ± 53.2	0.61 (0.23–0.8)	0.61 *
	HF	132.4 ± 54.7	129.8 ± 49.6	0.74 (0.5–0.86)	0.73 *
	Total	117 ± 54.4	117.3 ± 55.7	0.75 (0.64–0.83)	0.75 *

* *p* < 0.05. Abbreviations: PA = physical activity; Test 1 = results from first visit; Test 2 = results from second visit (after 1 week); Q_MPA = construct in questionnaire about moderate PA; Q_VPA = construct in questionnaire about vigorous PA; Q_MVPA = constructs in questionnaire about moderate and vigorous PA: Q = questionnaire; AC = accelerometer; ICC = intraclass correlation coefficient; 95% CI = 95% confidence interval.

**Table 4:** Criterion validity for each PA category of the SHAPES questionnaire.

	Spearman’s rho (AC vs. Q)			
	LF	IF	HF	Total
MPA	0.03	−0.12	0.34 *	0.10
VPA	0.10	0.42	0.70 *	0.51 *
MVPA	0.14	0.20	0.50 *	0.32 *

* *p* < 0.05. Abbreviations: MPA = moderate PA; VPA = vigorous PA; Q_MVPA = moderate and vigorous PA; AC = accelerometer; Q = questionnaire.

## Data Availability

Data supporting the reported results can be found in the supplementary SPSS file.

## Supplementary Materials

The following are available online at https://www.mdpi.com/article/10.3390/ijerph19031304/s1, Supplement File S1 SHAPES questionnaire, Supplement File S2: Instructions accelerometer.
