# Supplementary material for: Do Fitter Children Better Assess Their Physical Activity with Questionnaire Than Less Fit Children?
Source: Int J Environ Res Public Health. 2022 Jan 24;19(3):1304. doi: 10.3390/ijerph19031304 (PMC8835444; doi:10.3390/ijerph19031304)
Supplement: Supplementary file 1 [file ijerph-19-01304-s001.zip › Supplementary 01_SHAPES questionnaire.pdf]

Q1 - Are you male or female?

- ◯ Male
- ◯ Female

Q2 – Your date of birth

PHYSICAL ACTIVITY

HARD physical activities are jogging, team sports, fast dancing, jump-rope and any other physical activities that increase your heart rate and make you breathe hard and sweat.

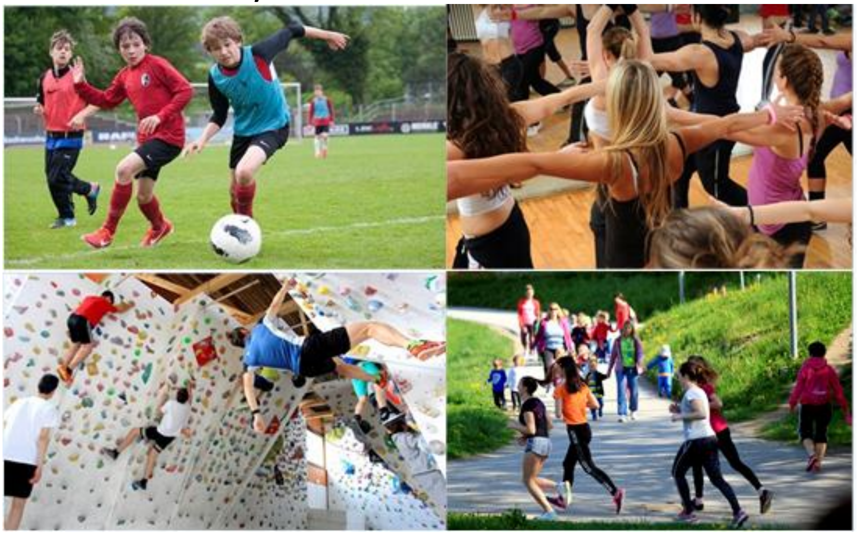

MODERATE physical activities are lower intensity activities such as walking, biking to school, and recreational swimming.

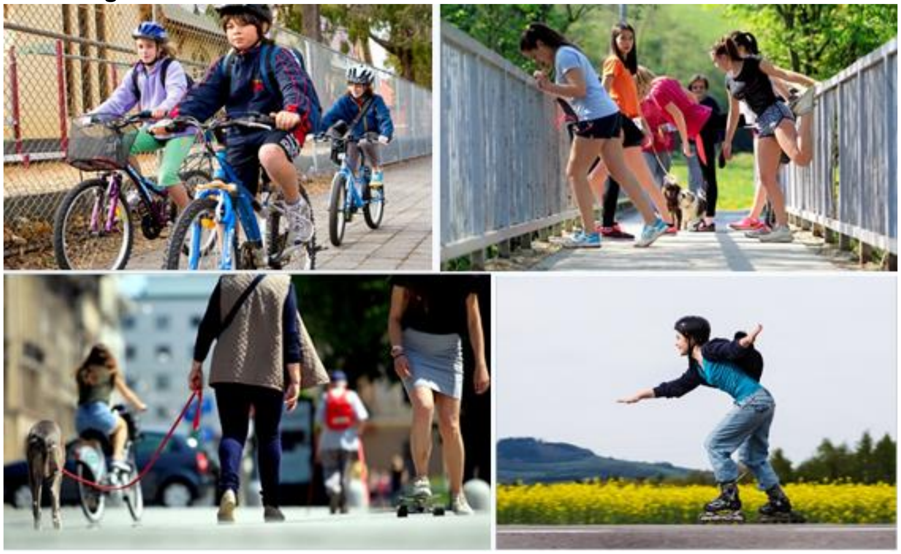

Q3 - Mark how many minutes of HARD physical activity you did in the last 7 days. This includes physical activity during physical education class, lunch, recess, after school, evenings, and spare time.

For example: if you did 1 hour and 15 minutes of hard activity on Monday, you will need to fill in the 1 hour circle and the 15 minute circle, as shown below:

	Hours				Minutes			
	0	1	2	3	0	15	30	45
Monday	○	◉	○	○	○	◉	○	○

. Hours

	0	1	2	3
Monday	○	○	○	○
Tuesday	○	○	○	○
Wednesday	○	○	○	○
Thursday	○	○	○	○
Friday	○	○	○	○
Saturday	○	○	○	○
Sunday	○	○	○	○

Minutes

	0	15	30	45
Monday	○	○	○	○
Tuesday	○	○	○	○
Wednesday	○	○	○	○
Thursday	○	○	○	○
Friday	○	○	○	○
Saturday	○	○	○	○
Sunday	○	○	○	○

Q4- Mark how many minutes of MODERATE physical activity you did in the last 7 days. This includes physical activity during physical education class, lunch, recess, after school, evenings, and spare time

For example: if you did 1 hour and 30 minutes of moderate activity on Monday, you will need to fill in the 2 hour circle and the 45 minute circle, as shown below.

	Hours				Minutes			
	0	1	2	3	0	15	30	45
Monday	○	◉	○	○	○	○	◉	○

Hours

	0	1	2	3
Monday	○	○	○	○
Tuesday	○	○	○	○
Wednesday	○	○	○	○
Thursday	○	○	○	○
Friday	○	○	○	○
Saturday	○	○	○	○
Sunday	○	○	○	○

Minutes

	0	15	30	45
Monday	○	○	○	○
Tuesday	○	○	○	○
Wednesday	○	○	○	○
Thursday	○	○	○	○
Friday	○	○	○	○
Saturday	○	○	○	○
Sunday	○	○	○	○
