# Supplementary material for: Do Fitter Children Better Assess Their Physical Activity with Questionnaire Than Less Fit Children?
Source: Int J Environ Res Public Health. 2022 Jan 24;19(3):1304. doi: 10.3390/ijerph19031304 (PMC8835444; doi:10.3390/ijerph19031304)
Supplement: Supplementary file 1 [file ijerph-19-01304-s001.zip › Supplementary 02.pdf]

INSTRUCTIONS FOR USE OF THE RM32 ACCELEROMETER FOR MEASURING PHYSICAL ACTIVITY

The RM32 accelerometer evaluates physical activity based on accelerations and poses no danger to your organism. If you want to get a credible feedback about your body activity, you need to wear the accelerometer 24 hours a day for 7 days - even during sleep. Take off the accelerometer only when showering and doing activities in water, such as swimming, and then put it back on according to the instructions. It is very important that you are physically active as ussual week, otherwise the feedback will be wrong! At the end of each day, you will also need to fill out a diary briefly describing your daily activity. This will help us understand your activity and improve the information we will give you after the measurements.
The accelerometer was cleaned and the band was washed before we attached it to your waist.

Instructions for using the accelerometer
The accelerometer must be attached to the front right side of the belt (Figure S1). It is worn 24 hours a day, except for water activities, and turns on by itself so you do not have to press any buttons.

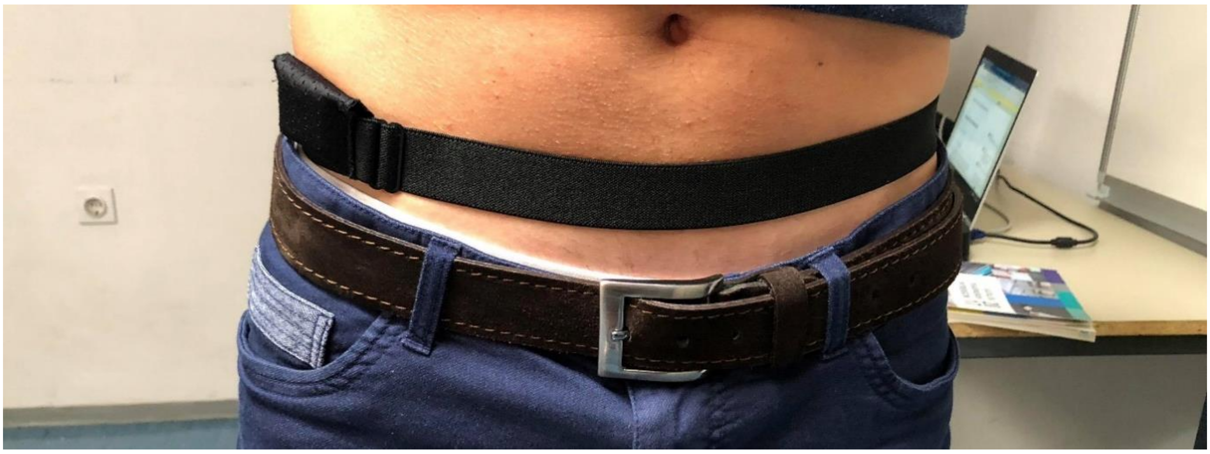

Figure S1: During the day, the accelerometer must be attached to a long strip on the front right side of the belt.

Before installation, the accelerometer must be turned so that the charging slot (Figure S2 left) faces the ground and the opening of the pouch on the conveyor belt faces up. The bag containing the accelerometer and the strap that closes it face away from the body (this avoids skin contact and possible faster accidental opening of the bag).

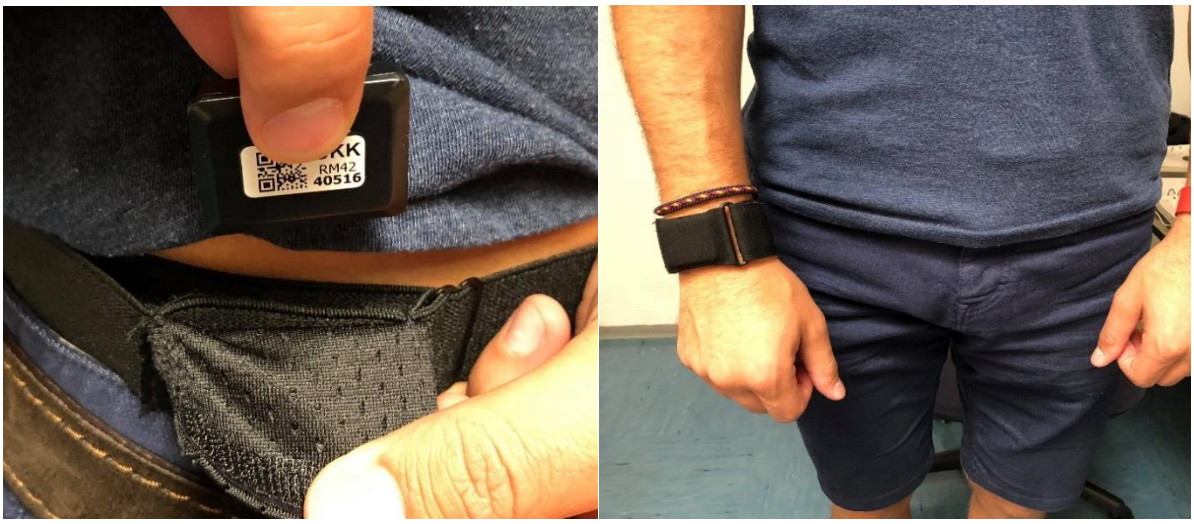

Figure S2: Changing the meter from the long to the short strap.

During sleep, the accelerometer is worn on the right wrist (Figure S2 right) - slide the accelerometer into the short elastic band.

WHAT FEEDBACK ARE YOU GOING TO GET ?

We will analyse the intensity and amount of your daily physical activity on different days of the week. This includes low, moderate, and vigorous physical activity, sitting, lying down, and sleeping. The data can help you understand your lifestyle and alert you that intervention may be necessary. For the appropriate feedback, it is very important that you wear the accelerometer according to the instructions and log your activities. The analysis of the data is and will be done with the help of Finnish partners, so we will be available to you about 2 months after the measurements. For more information about wearing accelerometers, please contact us at the e-mail address vedrana.sember@fsp.uni-lj.si.
